# Interbase-FRET binding assay for pre-microRNAs

**DOI:** 10.1038/s41598-021-88922-0

**Published:** 2021-04-30

**Authors:** Mattias Bood, Anna Wypijewska del Nogal, Jesper R. Nilsson, Fredrik Edfeldt, Anders Dahlén, Malin Lemurell, L. Marcus Wilhelmsson, Morten Grøtli

**Affiliations:** 1 Department of Chemistry and Molecular Biology, University of Gothenburg, 412 96 Gothenburg, Sweden; 2 Department of Chemistry and Chemical Engineering, Chemistry and Biochemistry, Chalmers University of Technology, 412 96 Gothenburg, Sweden; 3 Medicinal Chemistry, Research and Early Development, Cardiovascular, Renal and Metabolism (CVRM), BioPharmaceuticals R&D, AstraZeneca, Gothenburg, Pepparedsleden 1, 431 83 Mölndal, Sweden; 4 Structure & Biophysics, Discovery Sciences, BioPharmaceuticals R&D, AstraZeneca, Gothenburg, Pepparedsleden 1, 431 83 Mölndal, Sweden; 5 Oligonucleotide Discovery, Discovery Sciences, BioPharmaceuticals R&D, AstraZeneca, Gothenburg, Pepparedsleden 1, 431 83 Mölndal, Sweden

**Keywords:** Analytical chemistry, Medicinal chemistry

## Abstract

The aberrant expression of microRNAs (miRs) has been linked to several human diseases. A promising approach for targeting these anomalies is the use of small-molecule inhibitors of miR biogenesis. These inhibitors have the potential to (i) dissect miR mechanisms of action, (ii) discover new drug targets, and (iii) function as new therapeutic agents. Here, we designed Förster resonance energy transfer (FRET)-labeled oligoribonucleotides of the precursor of the oncogenic miR-21 (pre-miR-21) and used them together with a set of aminoglycosides to develop an interbase-FRET assay to detect ligand binding to pre-miRs. Our interbase-FRET assay accurately reports structural changes of the RNA oligonucleotide induced by ligand binding. We demonstrate its application in a rapid, qualitative drug candidate screen by assessing the relative binding affinity between 12 aminoglycoside antibiotics and pre-miR-21. Surface plasmon resonance (SPR) and isothermal titration calorimetry (ITC) were used to validate our new FRET method, and the accuracy of our FRET assay was shown to be similar to the established techniques. With its advantages over SPR and ITC owing to its high sensitivity, small sample size, straightforward technique and the possibility for high-throughput expansion, we envision that our solution-based method can be applied in pre-miRNA–target binding studies.

## Introduction

MicroRNAs (miRs) are short (typically 22 nt) single-stranded RNAs that have important cellular functions and are dysregulated in a variety of human diseases, including cancers, viral infections, cardiovascular disease, and inflammatory diseases^1^. Currently, 1,917 human miR precursor genes, which are processed into 2,675 mature miRs, have been annotated in the miRBase database^2^ and are thought to regulate one-third of the human proteome^3^. Genes encoding miRs are transcribed as long RNA precursors, which are processed by the Drosha nuclear microprocessor complex to yield approximately 70 nt hairpin precursor miRs (pre-miRs)^4^. Pre-miRs are then transported to the cytoplasm for further processing by RNase III Dicer to form a double-stranded RNA (dsRNA) molecule. One of its strands, the mature miR, is loaded into the RNA-induced silencing complex, which binds the complementary mRNA target through Watson–Crick base pairing and mediates gene silencing. Studies have demonstrated that numerous miRs act as tumor suppressors or oncogenes (also termed ‘onco-miRs’)^5^. Only a few onco-miRs have been well characterized so far, one of these being miR-21^6^. The identification of small molecules that bind to onco-miR precursors (such as the pre-miR-21 presented herein) could be a viable approach to inhibit the biogenesis of miRs that are involved in cancer development and could lead to mechanistically novel cancer therapies^7^. Furthermore, miRs have now been thoroughly validated as a therapeutic target^8^.

The most commonly employed method to target disease-associated miRs is based on the use of antisense technologies^9,10^. However, this approach primarily relies on modified oligonucleotide structures; these structures suffer from poor cell permeability and cellular distribution due to their intrinsic anionic character^11^. In contrast, small molecules offer the advantage of having good absorption, distribution, and oral bioavailability. Furthermore, rather than using sequence complementarity for binding, small molecules have the potential to recognize and bind to pre-miRs through RNA secondary or tertiary structural motifs, such as bulges, internal loops, hairpin loops, junctions, pseudoknots, or higher-order structural elements^12^.

Several cellular and non-cellular reporter-based assays have previously been applied to identify small-molecule inhibitors of pre-miR-mediated gene silencing^7^. Unfortunately, these assays all have their limitations. In general, small-molecule microarrays^13,14^, molecular beacons^15–17^, fluorescence polarization^18,19^, and catalytic enzyme-linked click chemistry assays^20^ all utilize reporter probes that are either large or amphiphilic and potentially interfere with the assay and/or are distant from the binding site of interest. Small-molecule binding assays, such as fluorescence indicator displacement^21^ require either selectively binding fluorophores or the displacement of more promiscuous probes, such as ethidium bromide, which can cause false negatives if the mode of binding differs. The hits from such assays have typically been verified by examining the maturation level of the pre-miR after adding the Dicer enzyme^16^ and in several cases they have been furthered characterized using additional biophysical techniques such as isothermal titration calorimetry (ITC)^13^, differential scanning calorimetry (DSC)^14^ or surface plasmon resonance (SPR)^22^.

Biophysical techniques such as ITC and SPR are well suited for resolving direct pre-miR engagement and were employed as benchmark methods in the current study. A significant advantage of ITC is that it can be readily applied to almost any RNA–ligand complex without the need to label both molecules. Additionally, it can be performed under a broad range of pH values, temperatures, and ionic concentrations. The major drawbacks of ITC, namely, the limited throughput and a significant sample size, is partly mitigated by SPR. However, SPR also has disadvantages, as it cannot easily discriminate between specific and non-specific interactions with the sensor surface.

Förster resonance energy transfer (FRET) is a non-radiative process in which energy is transferred between donor and acceptor chromophores^23^. The efficiency of this process is highly dependent on both distance and orientation between the chromophores. Consequently, information about the relative position of different donor- and acceptor-labelled biomolecules and thereby the structure of the biomolecule can be obtained^24^. FRET has been used in the molecular beacon (MB) assay format to measure the inhibition of Dicer-mediated pre-miR hairpin cleavage^15–17^. The compounds tested in previous studies have typically been ranked relative to each other using the fluorescence increase resulting from the Dicer RNase activity. A potential drawback of MB pre-miR-assays is that the large amphiphilic FRET probes are attached to the ends of the oligoribonucleotide; this can significantly impact the results because Dicer recognizes both the 3′- and 5′-end of the pre-miR^15,25^. Unfortunately, no counter-screen has been performed to determine the detailed binding parameters; thus, the developed MB assay could not be benchmarked against established techniques. While a FRET MB assay can identify hits for Dicer inhibition, it cannot resolve subtle changes in ligand binding, such as local conformational changes in the RNA structure^26^.

**Figure 1:**
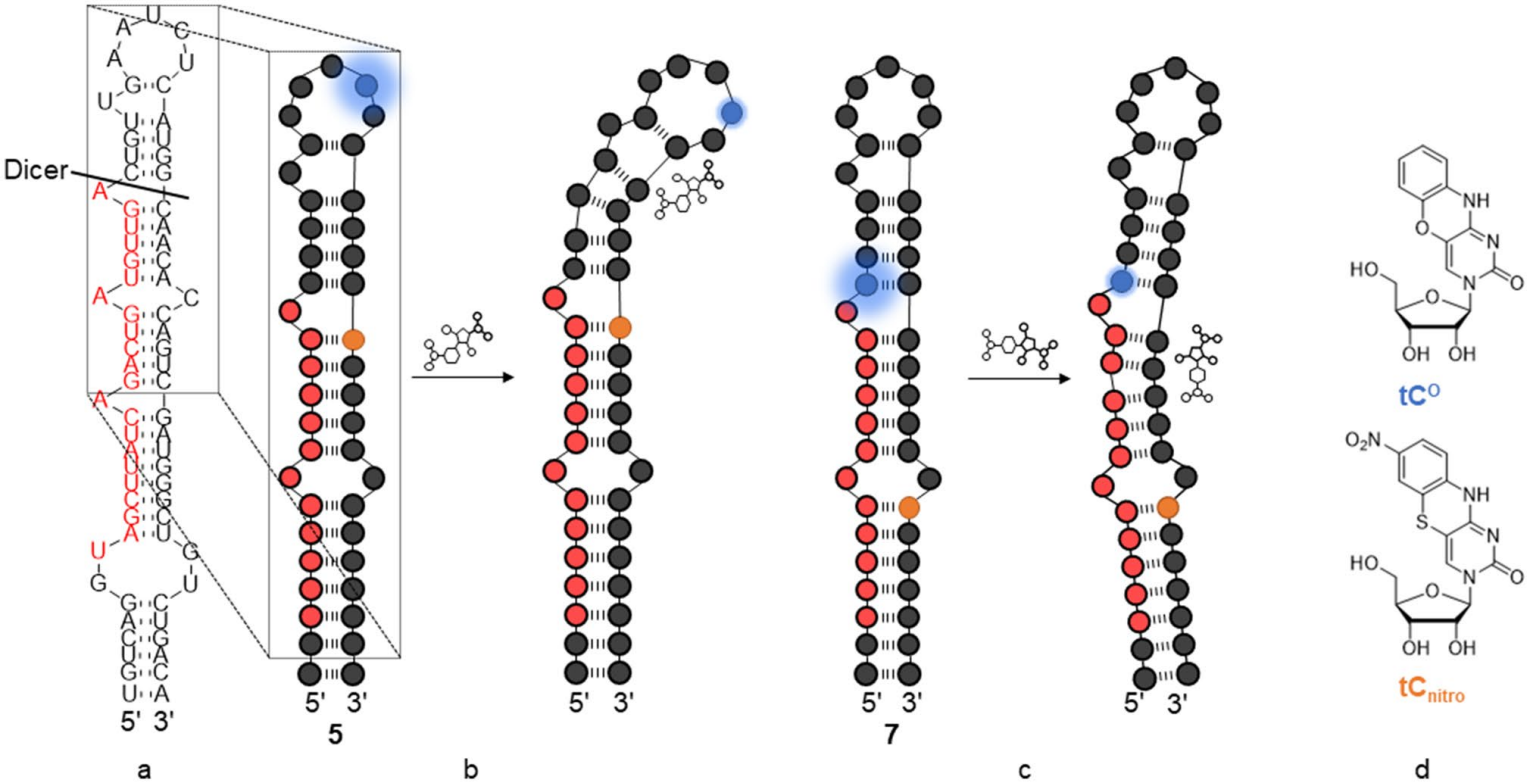
Design of the interbase-FRET assay. (**a**) The full-length pre-miR-21 (**1**) with miR-21 sequence in red and Dicer cleavage sites marked by the black line. (**b**) Ligand binding to the hairpin loop region of FRET-labelled pre-miR-21 construct (**5**, Table 1) causes a distance and/or orientation change between the fluorescent FRET donor (blue) and the non-fluorescent FRET acceptor (orange), thereby changing the FRET efficiency, and consequently, either an increase or a decrease in emission is observed. (**c**) Ligand binding to the stem FRET-labelled pre-miR-21 construct (**7**, Table 1). (**d**) The chemical structures of the fluorescent FRET donor tC^O^ (blue) and the non-fluorescent FRET acceptor tC_nitro_ (orange).

In an attempt to combine a high-information screening technique with a potential high-throughput screening method, we aimed to mitigate the shortcomings of MB assays and direct pre-miR engagement assays such as SPR. FRET between conformationally restricted, non-perturbing, fluorescent nucleobase analogs (FBAs), called interbase FRET, can be utilized to measure distance and orientation between the involved donor and acceptor molecules^27^. This offers the possibility not only to measure Dicer inhibition more accurately from a ligand-binding event due to their non-perturbing nature but could also, if positioned wisely, be used to identify site-selective ligand binders. We have previously demonstrated that the emissive cytosine analogue 3-(β-d-ribofuranosyl)-3H-benzo[b]pyrimido[4,5-e][1,4]oxazin-2(10H)-one (tC^O^)^39^ (Fig. 1d) can be used together with the non-emissive 3-(β-d-ribofuranosyl)-7-nitro-3H-pyrimido[5,4-b][1,4]benzothiazin-2(10H)-one (tC_nitro_)^28^ (Fig. 1d), to create an interbase-FRET pair. This combination of cytosine analogues have been used to monitor the transition from A- to Z-form RNA with no significant perturbation of the natural RNA structure^28^.

Previous work has demonstrated that aminoglycosides^29^ have a general affinity towards RNA^30–33^, including pre-miR-21^13,22,34–38^. However, the specificity of these binding events has yet to be fully elucidated^38^. Aminoglycosides have also been used to construct bifunctional aminoglycoside conjugates that consist of a pre-miRNA binding unit that is connected by a linker to a Dicer inhibiting unit that binds pre-miR-21 and regulates miR-21 maturation^19^. From the typical molecular structures of aminoglycosides (Figure S2), they are expected to be non-fluorescent and not to absorb light in the spectral region relevant for tC^O^–tC_nitro_ FRET (i.e., above 350 nm, Figure S1)^28,39^. Hence, aminoglycosides will not interfere with the energy transfer process of our FRET pair, and we therefore decided to employ them as test compounds in this work to validate our pre-miR-21 FRET-based ligand-binding assay, which utilizes a tC^O^–tC_nitro_ RNA FRET pair^28^.

Small molecules contained in screening libraries can themselves be fluorescent or act as a quencher, leading to potential false results through interference with the tC^O^–tC_nitro_ RNA FRET pair. However, this potential problem can be reduced by eliminating library compounds with larger conjugated aromatic systems. Furthermore, counter assays can be utilized to identify compounds that can potentially interfere with the detection method.

## Results and discussion

### FRET-labelled oligoribonucleotide design

Mature miRs do not contain secondary structural elements that allow specific and high-affinity binding by small molecules. Consequently, miR-regulating strategies utilizing small molecules are often based on pri- or pre-miR biogenesis. The pre-miRs are approximately 70 nt-long hairpins containing discrete secondary and tertiary structures that are susceptive to specific binding by drug-like molecules. An examination of the predicted secondary structure of the full-length pre-miR-21 hairpin (**1**, Table 1, Fig. 1a)^40^ and the available solution-NMR structure of the pre-miR-21 hairpin segment^34^ revealed that the majority of the secondary structures to which a ligand could potentially bind (mismatch bulges and a hairpin loop) were located in proximity to the hairpin loop and the functional Dicer processing site (Fig. 1a). From a chemical synthesis point of view, long, modified RNA sequences are challenging to prepare, which led us to truncate the full-length pre-miR-21 sequence to a 39 nt hairpin. We next added two extra GC pairs to mitigate end-fraying effects, yielding the 43 nt truncated pre-miR-21 oligoribonucleotide (**2**, Table 1). This construct was used as a starting point for the development of FRET-labelled oligoribonucleotides (**5** and **7**, Table 1, Fig. 1b,c) and corresponding donor-only oligoribonucleotides (**6** and **8**, Table 1) for our interbase-FRET assay. Because of this oligoribonucleotide design, our assay has the potential to probe structural changes within pre-miRs using a highly sensitive fluorescence-based readout (Fig. 1b,c).

**Table 1:** Sequences of oligoribonucleotides used in this study.

ID	Oligoribonucleotide sequence^a^
**1**	5ʹ-UGU CGG GUA GCU UAU CAG ACU GAU GUU GAC UGU UGA AUC UCA UGG CAA CAC CAG UCG AUG GGC UGU CUG ACA-3ʹ
**2**	5ʹ-CCG ACU GAU GUU GAC UGU UGA AUC UCA UGG CAA CAC CAG UCG G-3ʹ
**3**	5ʹ-biot-C6-CCG ACU GAU GUU GAC UGU UGA AUC UCA UGG CAA CAC CAG UCG G-3ʹ
**4**	5ʹ-biot-C6-CCG ACU GAU GUU GA**X** UGU UGA AU**Y** UCA UGG CAA CAC CAG UCG G-3ʹ
**5**	5ʹ-CCG ACU GAU GUU GAC UGU UGA AU**X** UCA UGG **Y**AA CAC CAG UCG G-3ʹ
**6**	5ʹ-CCG ACU GAU GUU GAC UGU UGA AU**X** UCA UGG CAA CAC CAG UCG G-3ʹ
**7**	5ʹ-CCG ACU GAU GUU GA**X** UGU UGA AUC UCA UGG CAA CAC **Y**AG UCG G-3ʹ
**8**	5ʹ-CCG ACU GAU GUU GA**X** UGU UGA AUC UCA UGG CAA CAC CAG UCG G-3ʹ

^a^Biot-C6 denotes 6-(5-((3a*S*,4*S*,6a*R*)-2-oxohexahydro-1*H*-thieno[3,4-d]imidazol-4-yl)pentan-amido)hexyl phosphate. **X** denotes the fluorescent FRET donor tC^O^ and **Y** denotes the non-emissive FRET acceptor tC_nitro_. Oligoribonucleotide **1** is the full-length pre-miR-21. Oligoribonucleotide **2** is unmodified 43 nt pre-miR-21 hairpin used in ITC experiments. Oligoribonucleotide **3** is biotin-labelled unmodified 43 nt pre-miR-21 hairpin used in SPR experiments. Oligoribonucleotide **4** is biotin-labelled FRET pair modified 43 nt pre-miR-21 hairpin used in SPR. Oligoribonucleotides **5**–**8** are FRET pair or FRET donor modified 43 nt pre-miR-21 hairpins used in FRET experiments.

We envisioned that by replacing a pair of C residues in oligoribonucleotide **2** with the tC^O^–tC_nitro_ RNA FRET pair (Fig. 1d), we would be able to monitor changes in the FRET efficiency that arise from ligand binding (Fig. 1b,c). Furthermore, using sequences **5** and **7** in parallel (which report on different regions of the pre-miR-21) also enables us to identify where the ligand binding takes place. Based on previous work regarding the tC^O^–tC_nitro_ inside the RNA duplexes^28^, we anticipated that inserting this FRET pair would have a minimal effect on the pre-miR-21 structure. The corresponding 5′-biotinylated sequences (**3** and **4**, Table 1), enabling attachment of the oligoribonucleotide to the streptavidin-coated surface, were prepared for immobilisation onto a streptavidin-coated SPR chip. Sequence **3** contains no donor or acceptor while sequence **4** contains the tC^O^-tC_nitro_ FRET pair (Table 1) to verify if the incorporation of our labelles has an impact on ligand binding readout when using the SPR technique.

### ITC and SPR interaction reference measurements

**Figure 2:**
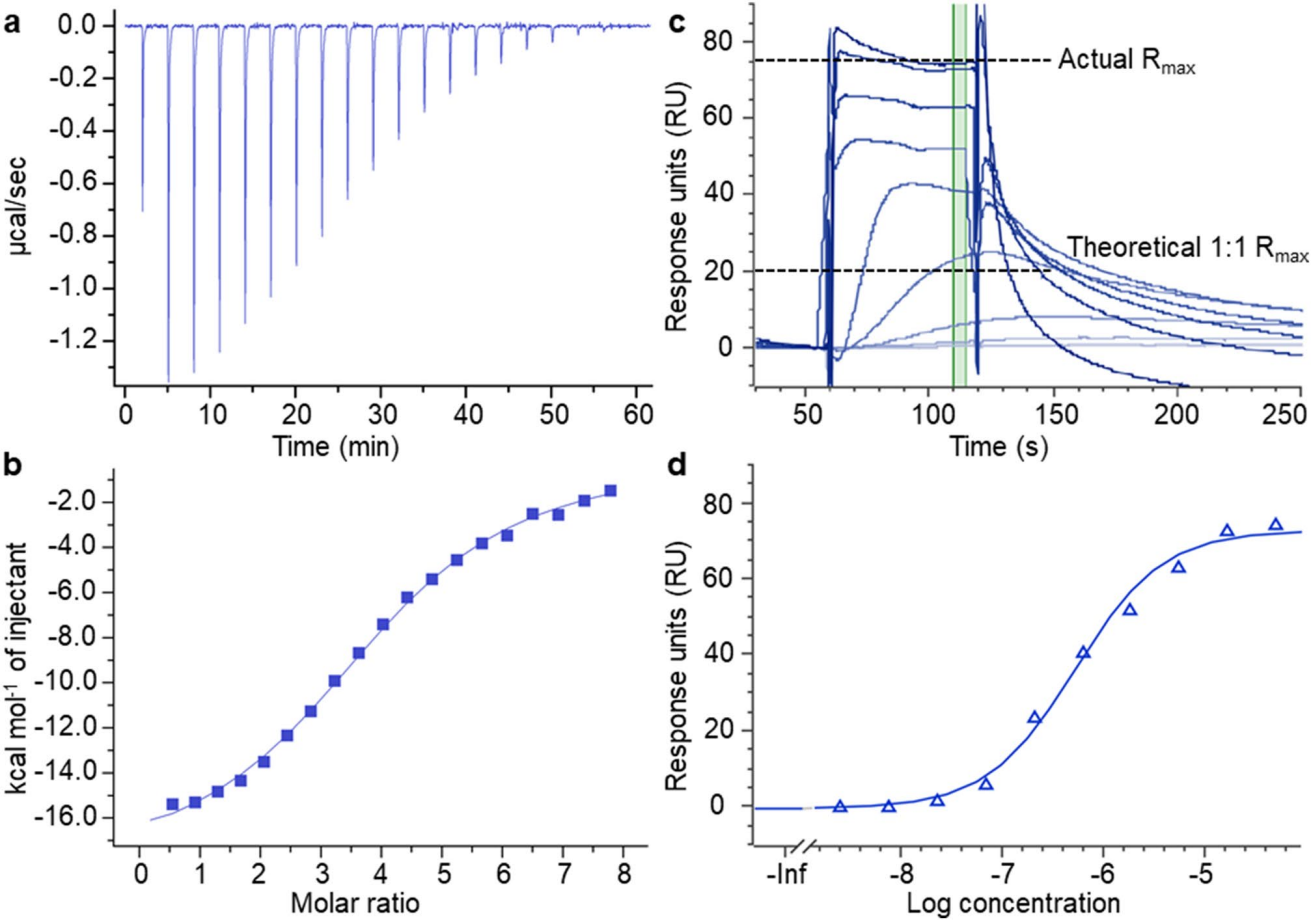
Neomycin binding to pre-miR-21. (**b**) ITC data showing heat evolution upon titrating neomycin to oligoribonucleotide **2** (10 µM). (**b**) Integrated peak areas from panel (**a**) versus neomycin:**2** molar ratio for the successive additions and the fitted curve (solid line, assuming a 1:1 binding) yielding a *K*_d_ of 5.2 ± 0.8 µM. (**c**) Neomycin binding to oligoribonucleotide **3** at 10 different concentrations using SPR, where the green marked region shows steady-state conditions from which *K*_d_ values are determined. (**d**) The fitted curve (solid line, assuming an equivalent affinity for all binding sites) from panel (**c**) yielding an average *K*_d_ of 3.9 ± 2.8 µM.

ITC was used to explore experimental conditions for the aminoglycoside neomycin binding to unmodified truncated pre-miR-21 (**2**, Table 1), including optimization of the buffer conditions. Ultimately, we employed a sodium cacodylate buffer (20 mM sodium cacodylate, 80 mM NaCl, 100 mM total Na^+^, pH 7.2) that was previously used in studies of aminoglycosides binding to the HIV-1 RNA dimerization initiation site^41^. This buffer resulted in excellent reproducibility of the ITC binding isotherms and a dissociation constant (*K*_d_) of 5.2 ± 0.8 µM for the interaction between pre-miR-21 **2** and neomycin (Fig. 2a,b).

To investigate whether the tC^O^–tC_nitro_ RNA FRET pair had any impact on the binding affinity between aminoglycosides and our FRET-labelled oligoribonucleotides, a comparative SPR measurement was performed using the biotinylated, FRET pair-containing pre-miR-21 (**4**, Table 1) and its FRET pair-free counterpart (**3**, Table 1). The *K*_d_ values of neomycin for sequences **3** and **4** were determined to be 3.9 ± 2.8 µM (Fig. 2c,d) and 2.7 ± 1.7 µM (Table 2), respectively. These results agree well with the ITC data (Fig. 2a,b). The difference in affinity of neomycin for **3** and **4** was minor and within the experimental error of the SPR measurements. This strongly suggests that the FRET pair does not perturb the overall secondary structure of the pre-miR-21 construct nor the ligand–pre-miR interaction.

**Table 2:** SPR binding affinity for oligoribonucleotides **3** and **4**, interbase-FRET binding affinity for oligoribonucleotides **5** and **7** and the respective rankings of all aminoglycosides.

SPR				Interbase-FRET			
Aminoglycoside	*K*_d_ [µM] of **3**	*K*_d_ [µM] of **4**	Δ*FRET*_15µM_ of **5**	Δ*FRET*_15µM_ of **7**	Averaged Δ*FRET*_norm_^a^	SPR Rank^b^	FRET Rank
Neomycin	4 ± 3	3 ± 2	0.042	0.045	0.97	1	1
Sisomicin	4 ± 2	4 ± 2	0.023	0.041	0.68	2	3
Tobramycin	8 ± 4	8 ± 5	0.024	0.049	0.77	3	2
Amikacin	15 ± 4	14 ± 3	0.021	0.020	0.49	4	8
Gentamicin	16 ± 1	13 ± 3	0.011	0.040	0.64	5	6
Apramycin	22 ± 12	23 ± 12	0.017	0.037	0.67	6	4
Netilmicin	28 ± 35	27 ± 34	0.024	0.045	0.66	7	5
Kanamycin A	44 ± 18	45 ± 23	0.009	0.041	0.52	8	7
Ribostamycin	90 ± 73	78 ± 67	0.006	0.024	0.33	9	10
Geneticin	101 ± 44	94 ± 57	0.010	0.027	0.43	10	9
Streptomycin	134 ± 76	115 ± 80	0.004	0.008	0.13	11	11
Hygromycin	291 ± 91	291 ± 110	0.001	0.000	0.01	12	12

^a^Averaged Δ*FRET*_norm_ for the two FRET-labelled oligoribonucleotides (**5** and **7**) was calculated as an average of their Δ*FRET*_norm_ values. The latter ones were obtained by dividing the Δ*FRET* from adding aminoglycoside to reach the total concentration of 15 µM to 1 µM oligoribonucleotide by the Δ*FRET* from adding aminoglycoside to reach 90% degree of complexation of the same oligoribonucleotide (Eq. 3, Tables S4-S5). ^b^SPR rank was determined from the average dissociation constant of oligoribonucleotides **3** and **4.**

### Dissociation constants of the pre-miR-21-aminoglycoside interaction determined using SPR

To benchmark our interbase-FRET binding assay (vide infra), we investigated the binding affinities of a series of 12 aminoglycosides (Tables 2 and S2; see Figure S2 for structures) to pre-miR-21 **3** and **4** using SPR. The obtained dissociation constants ranged from approximately 3 μM to 300 μM for both pre-miR-21 **3** and **4**, and these values were used to determine the SPR binding rank (Table 2). Because of the similar binding affinities for pre-miR-21 **3** and **4**, we concluded that the introduction of our FRET pair did not significantly alter the mode or strength of binding or the overall interaction between pre-miR and this type of ligand. Our results agree with a previous study by Yan et al. in which a fluorescence polarization assay was used to identify neomycin, netilmicin, sisomicin, and tobramycin as particularly potent binders to pre-miR-21^19^. Notably, the aminoglycosides exhibited a binding stoichiometry that varied between 4 and 5 depending on the aminoglycoside used, indicating that they bind non-specifically across pre-miR-21. Nearly identical binding stoichiometries were obtained for both **3** and **4** (unlabeled vs. FRET-labeled, respectively) (Table S3).

### Interbase-FRET binding assay design

Our FRET-based assay for studying small-molecule binding to pre-miRs is based on evaluating the integrated emission of the FRET pair-labeled (tC^O^ and tC_nitro_ incorporated, donor–acceptor, DA) pre-miR sequence before and after addition of the ligand in relation to the same being performed for the FRET donor-containing (tC^O^ incorporated, donor only, D) pre-miR sequence (see Materials and Methods Eqs. 1–4). The ligand is added to the FRET-labeled pre-miR, and the change in emission for the DA strand relative to the corresponding D strand is measured. The FRET efficiency is calculated as the fraction of the integrated emission from the DA and D sequences (Eq. 1). The difference in FRET before and after ligand addition (Δ*FRET*, Eq. 2) is then normalized between the two different ligand binding experiments (vide infra) to obtain Δ*FRET*_norm_ (Eq. 3, Tables S4 and S5). The average Δ*FRET*_norm_ for the two different sets of FRET-labeled oligoribonucleotides (averaged Δ*FRET*_norm_, Eq. 4) is used to rank the ligands (FRET rank), ranging from potent to less potent binders for the screened set of small molecules (Tables 2 and S6).

### Aminoglycoside affinity rank for pre-miR-21 determined by interbase-FRET assay

To obtain a significant change in FRET, the aminoglycosides (Table 2 and Figure S2) were added to sequences **5**–**8** (Table 1) at a concentration corresponding to a 90% degree of pre-miR-21 complexation. Higher degree of complexation would require non-feasible concentrations or volumes of the aminoglycoside stock solutions. The hairpin loop-labeled oligonucleotide (**5**, Table 1) showed a range of 2–5% with a 3% average increase in FRET efficiency (Figure S4), whereas the stem-labeled oligonucleotide (**7**, Figure S6) showed an average increase of 5% (except for hygromycin, which induced a decrease in FRET efficiency upon binding; Figure S6). For the donor-only control sequences (**6** and **8**, Table 1) small changes in FRET efficiency were observed (Figure S5 and S7), indicating that the local microenvironment of the donor changes upon the addition of aminoglycosides. Taken together, the results indicate that the aminoglycosides bind to the hairpin loop and to the stem parts of the FRET-labeled pre-miR constructs (**5** and **7**) and cause only small changes in the relative orientation/distance of the donor and acceptor. Furthermore, these results indicate that none of the aminoglycosides bind selectively to the hairpin loop or the stem parts of the labeled pre-miR constructs (**5** and **7**) but rather across the whole pre-miR. This is in agreement with the aminoglycoside:oligoribonucleotide binding ratio of 4–5:1 measured by SPR. It is also in line with work from the groups of Arenz and Maiti, showing that aminoglycoside antibiotics, such as kanamycin and streptomycin, generally inhibit Dicer-mediated miRNA processing by non-specific binding to the Dicer substrate, the pre-miR^15,16^.

The second set of ligand-binding experiments was designed to investigate whether our FRET-based assay could distinguish relative binding affinities of the aminoglycosides (Table 2, Figure S2). Furthermore, we wanted to investigate if Δ*FRET* for the aminoglycosides would correlate with the SPR affinity values.

Aminoglycosides were added to 1 µM FRET-labeled pre-miRs (**5** and **7**; Table 1) and their corresponding donor-only references (**6** and **8**; Table 1) at a total aminoglycoside concentration of 15 µM, chosen from SPR *K*_d_ as a middle-point between potent and non-potent binders (Table 2, Figure S8-S11). The data clearly show that high-affinity aminoglycosides such as neomycin (avg. SPR *K*_d_ = 3.3 µM, Table 2) caused a relatively large change in emission (avg. Δ*FRET* = 0.043 for **5** and **7**, Table 2). By contrast, aminoglycosides with lower affinity such as streptomycin (avg. SPR *K*_d_ = 124 µM, Table 2) caused minimal changes in emission (avg. Δ*FRET* = 0.006 for **5** and **7**, Table 2, for further details see Table 2). Overall, this experiment enabled us to distinguish the most potent binders with Δ*FRET* of 0.02–0.04 from the less potent binders with Δ*FRET* of 0.000–0.02.

By comparing the averaged Δ*FRET*_norm_ values based on both types of FRET experiments (adding aminoglycoside to reach a total concentration of 15 µM and adding aminoglycoside to reach a 90% degree of complexation; the latter representing the almost saturated binding event and hence a maximum change in FRET) and sorting them from high to low, the aminoglycosides were ranked based on FRET (Table 2, FRET Rank, Eq. 4). Overall, the FRET rank matched well with the SPR rank within a given class of ligand affinity (high, medium, and low), and the experimental errors of the two techniques were low. As in SPR, neomycin, sisomicin, and tobramycin were found to have the highest affinity for pre-miR-21 (avg. *K*_d_ < 10 µM, Table 2) using the FRET assay. Amikacin, gentamicin, apramycin, netilmicin, and kanamycin A all belong to the medium affinity group of pre-miR-21 ligands (avg. 15–45 µM, Table 2). A good correlation was observed when plotting the log *K*_d_ from SPR of **4** against the averaged Δ*FRET*_norm_ (R^2^ = 0.82, Figure S4). Finally, ribostamycin, geneticin, streptomycin, and hygromycin were found to be low-affinity binders in SPR (avg. *K*_d_ > 84 µM) and in our FRET assay. Thus, our interbase-FRET binding assay discriminates between high-, medium- and low-affinity binders equally well as the established SPR technique.

## Conclusion

In summary, we present a novel pre-miR binding assay based on interbase-FRET between a fluorescent tC^O^ donor and a non-fluorescent tC_nitro_ acceptor that incorporates into a pre-miR hairpin. Measuring the FRET efficiency changes enabled determination of the binding affinities between an onco-miR precursor (pre-miR-21, used here as a model pre-miR) and aminoglycoside antibiotics as small-molecule ligands. Initially, we used SPR to prove that the introduction of our FRET pair does not perturb the binding affinity. Importantly, changes in the FRET efficiency in our new established FRET-based assay correlated well with the *K*_d_ ranking of the aminoglycoside binding to pre-miR-21, as determined by the established SPR technique. We envisage that our assay will be useful for investigating ligand binding, including drug molecules and peptides, to various pre-miRs for the modulation of miR biogenesis and disease pathway modulation. Considering the low ligand and pre-miR amounts required and the sensitivity of our method, this assay should be compatible with high-throughput screening. We are currently investigating site-selective pre-miR binders using our novel interbase-FRET assay. Additionally, to increase its versatility, we have already expanded the RNA interbase-FRET methodology to include modifications of adenine positions^41^.

## Methods

### Synthesis of RNA oligonucleotides

The pre-miR-21 and truncated pre-miR-21 oligoribonucleotide sequences used in this study are listed in Table 1. Sequences **3** and **5**–**8** were purchased from ATDBio Ltd, Southampton, UK and Eurogentec, Liège, Belgium, respectively. Oligoribonucleotides **5** and **7** contained a FRET pair, while **6** and **8** contained a FRET donor only. Oligoribonucleotides **2** and **4** were synthesized in-house; see the Supplementary Information for details on their synthesis and characterization.

### General information on photophysical measurements and sample preparation

Sodium cacodylate buffer (20 mM sodium cacodylate, 80 mM NaCl, 100 mM total Na^+^, pH 7.2) was used for all measurements unless otherwise stated. Absorption spectra of the oligoribonucleotides were recorded between 200 and 550 nm on a Cary 4000 or Cary 5000 (Varian Technologies) at a scan rate of 600 nm/min and spectral bandwidth of 2.0 nm. The absorption at 260 nm was used to calculate the oligoribonucleotide concentration, and the molar absorptivity at 260 nm was taken as the linear combination of the molar absorptivities of the individual bases at this wavelength, multiplied by 0.9 to account for the effect of base stacking. The values used for the molar absorptivity of each base at 260 nm were: *ε*(T) = 9300 M^–1^ cm^–1^, *ε*(C) = 7400 M^–1^ cm^–1^, *ε*(G) = 11,800 M^–1^ cm^–1^, *ε*(A) = 15,300 M^–1^ cm^–1^, *ε*(tC^O^) = 11,000 M^–1^ cm^–1^, and *ε*(tC_nitro_) = 9700 M^–1^ cm^–1^. All spectroscopic measurements were carried out in a 0.3 cm pathlength quartz cuvette with a 45 µL chamber volume (Hellma). Pre-miR annealing was achieved by heating the solution rapidly (approximately 5–10 °C/min) to 90 °C, holding for 2.0 min, then cooling to 5.0 °C at approximately 1 °C/min. Aminoglycosides (Figure S2 in the Supporting Information) were purchased from various chemical vendors. Stock solutions (10 mM) were prepared gravimetrically and further diluted to the desired concentrations.

### Isothermal titration calorimetry (ITC) measurements

ITC was performed on a MicroCal iTC200 from Malvern Panalytical. A 206 µL aliquot of 10 µM oligoribonucleotide was loaded into the sample cell of the ITC using a gastight syringe. Aminoglycoside (100–300 µM) was added to the ITC titration syringe, and the cell was kept at 25 °C. The initial delay time was set to 120 s and the stirring speed of the syringe to 750 rpm. In each addition, a volume of 2.0 μL was injected over the course of 2.0 s from the titration syringe into the cell. The total number of additions was 20, administered with 180 s intervals. The aminoglycoside–oligoribonucleotide binding data were corrected for the heat of ligand and oligoribonucleotide dilution by subtracting the average heat per injection of aminoglycoside to buffer and buffer to oligoribonucleotide, respectively. The *K*_d_ was determined using the Origin-based MicroCal iTC200 instrument software by non-linear curve fitting to a 1:1 binding model.

### Surface plasmon resonance (SPR) measurements

Biotinylated oligoribonucleotide was immobilized on a streptavidin-coated biosensor chip (XanTec Bioanalytics GmbH). Capture levels were 300–500 response units. Each aminoglycoside sample was injected over the surface in a 10 concentration–response series. Sensorgrams were fitted with a steady-state model (region shown in Fig. 2c) using the SPR module of the Genedata Screener software package to obtain the *K*_d_ values and assuming equivalent affinity for all binding sites. The immobilization step was repeated to ensure a fresh surface prior to each run. The stoichiometry was obtained by dividing the observed maximum response (*R*_max_) by the theoretical *R*_max_. The theoretical *R*_max_ was obtained from the molecular weight ratio for each aminoglycoside vs. oligoribonucleotide multiplied by the total immobilization level.

### Fluorescence measurements and interbase-FRET binding assay

Steady-state fluorescence emission spectra were recorded on a Spex Fluorolog 3 (JY Horiba) with excitation at 365 nm and the excitation slit width set to 1.5 nm. The initial concentration of the oligoribonucleotide sample was 1.0 µM in all measurements, and the initial sample volume was 60 µL. Emissions were recorded between 380 and 700 nm at a scan rate of 600 nm min^–1^, with the emission slit width set to 5.0 nm. All spectra were recorded in duplicate, which were averaged prior to further evaluation.

For experiments where the aminoglycoside was added to reach a 90% degree of FRET-labeled oligoribonucleotide (**5**–**8**) complexation by the aminoglycoside, the dissociation constant obtained from SPR was used to calculate the required amount of aminoglycoside. SPR revealed a binding stoichiometry of 4–5:1 (aminoglycoside:oligoribonucleotide). However, owing to the simple binding dynamics observed (Fig. 2d), as well as nearly saturating the oligoribonucleotide with the aminoglycoside before measuring the emission spectrum, a 1:1 binding model could be used.

Reaching a 90% degree of FRET-labeled oligoribonucleotide (**5**–**8**) complexation by the aminoglycoside was achieved by preparing 60 µL of the desired oligoribonucleotide at a concentration of 1 µM in a reduced volume quartz cuvette and adding 5 µL of varying concentrations of aminoglycoside solution (Table S7). Emission spectra of the FRET-labeled oligoribonucleotide (**5** and **7**) were recorded and compared before and after ligand addition. This procedure was then repeated for the corresponding oligoribonucleotide containing only the FRET donor (**6** and **8**, Table 1).

For experiments where the aminoglycoside was added to reach a final concentration of 15 µM in the cuvette, 60 µL of 1 µM oligoribonucleotide was mixed with 1 µL of 915 µM aminoglycoside. An identical protocol to the one presented above was executed to obtain the emission spectra and calculate the FRET efficiency.

The averaged and normalized Δ*FRET* (averaged Δ*FRET*_norm_) resulting in the ranking presented in Table 2 was calculated by Eqs. (1–4), shown below.

The FRET efficiency (*E*) between the donor and acceptor within an oligoribonucleotide was calculated according to Eq. (1):

1 $$ E = 1 - \frac{{I_{{{\text{DA}}}} }}{{I_{{\text{D}}} }} $$

where *I*_D_ and *I*_DA_ are the integrated (over wavelength) steady-state emission spectra for the donor only and donor–acceptor samples, respectively. The change in FRET efficiency (Δ*FRET*) was calculated according to Eq. (2):

2 $$ {\Delta }FRET = E_{0} - E_{{\text{L}}} $$

where *E*_0_ and *E*_*L*_ are the FRET efficiencies before and after addition of the ligand, respectively. The normalized change in FRET efficiency (Δ*FRET*_norm_) was calculated according to Eq. (3):

3 $$ {\Delta }FRET_{{{\text{norm}}}} = \frac{{\Delta FRET_{{15{ }\,\upmu {\text{M}}}} }}{{\Delta FRET_{90\% } }} $$

where ${\Delta FRET}_{15\mathrm{ \mu M}}$ is the change in FRET efficiency upon adding 15 µM ligand, and ${\Delta FRET}_{90\%}$ is the change in FRET efficiency upon adding ligand at a concentration corresponding to a 90% degree of complexation (based on the *K*_d_ from SPR and assuming 1:1 binding). Finally, the ${\mathrm{Averaged }\Delta FRET}_{\mathrm{norm}}$ was calculated according to Eq. (4):

4 $$ {\text{Averaged}}\,{{ \Delta }}FRET_{{{\text{norm}}}} = \frac{{\left| {{\Delta }FRET_{{{\text{norm}},5}} } \right| + \left| {{\Delta }FRET_{{{\text{norm}},7}} } \right|}}{2} $$

where ${\Delta FRET}_{\mathrm{norm},5}$ and ${\Delta FRET}_{\mathrm{norm},7}$ are the normalized change in FRET efficiencies for oligoribonucleotides pairs **5** and **7**, respectively.

## Supplementary Information

Supplementary Information
