# Supplementary material for: Interbase-FRET binding assay for pre-microRNAs
Source: Sci Rep. 2021 Apr 30;11:9396. doi: 10.1038/s41598-021-88922-0 (PMC8087795; doi:10.1038/s41598-021-88922-0)
Supplement: Supplementary file 1 — Supplementary Information [file 41598_2021_88922_MOESM1_ESM.pdf]

**Supplementary Information for:**

# Interbase-FRET Binding Assay for pre-microRNAs

Mattias Bood,[1,3] Anna Wypijewska del Nogal,[2] Jesper R. Nilsson,[2] Fredrik Edfeldt,[4] Anders Dahlén,[5] Malin Lemurell,[3] L. Marcus Wilhelmsson,[2] and Morten Grøtli[1,*]

[1]Department of Chemistry and Molecular Biology, University of Gothenburg, SE-412 96 Gothenburg, Sweden
[2]Department of Chemistry and Chemical Engineering, Chemistry and Biochemistry, Chalmers University of Technology, Gothenburg, SE-412 96, Sweden
[3]Medicinal Chemistry, Research and Early Development, Cardiovascular, Renal and Metabolism (CVRM), BioPharmaceuticals R&D, AstraZeneca, Gothenburg, Pepparedsleden 1, Mölndal, SE-431 83, Sweden
[4]Structure & Biophysics, Discovery Sciences, BioPharmaceuticals R&D, AstraZeneca, Gothenburg, Pepparedsleden 1, Mölndal, SE-431 83, Sweden
[5]Oligonucleotide Discovery, Discovery Sciences, BioPharmaceuticals R&D, AstraZeneca, Gothenburg, Pepparedsleden 1, Mölndal, SE-431 83, Sweden

Correspondence and request for materials should be addressed to M.G. (grotli@chem.gu.se)

List of contents

Oligonucleotide synthesis, purification and analysis ........ S2
Supplementary figures and tables ........ S3
References ........ S12

**Oligonucleotide synthesis, purification and analysis**

Oligoribonucleotide **3** (Table 1, main text) was purchased from ATDBio Ltd, Southampton, UK. Oligoribonucleotides **5-8** were purchased from Eurogentec, Liège, Belgium. Oligoribonucleotides **2** and **4** were synthesised in-house on an ÄKTA OligoPilot Plus 10 synthesizer (GE Healthcare), on a 32 µmole scale, using a standard synthesis cycle of detritylation (3% dichloroacetic acid in toluene), coupling (coupling agent: 0.3 M 5-(benzylthiobenzylthio)-1*H*-tetrazole in acetonitrile), capping (Cap A: 20% *N*-methylimidazole and 80% acetonitrile; Cap B: 20% pyridine, 20% acetic anhydride and 60% acetonitrile) and oxidation (0.05 M iodine in pyridine and water), and pre-loaded solid supports (Primer Support 5G ~300 µmole/g, GE Healthcare). The $tC^{O}$- and $tC_{nitro}$-protected β-cyanoethyl phosphoramidites were prepared according to literature procedures.(1) All fully protected RNA *β*-cyanoethyl phosphoramidite monomers were dissolved in anhydrous acetonitrile (0.1 M) or in acetonitrile/toluene 1:3 ($tC_{nitro}$) under argon immediately prior to use. The phosphoramidite re-circulation time/coupling time for normal A, G, C and U monomers was 5 min and was extended to 20 min for $tC^{O}$ and $tC_{nitro}$. Stepwise coupling efficiencies and overall yields were determined by automated trityl cation absorption monitoring exceeding 96% for all oligoribonucleotides synthesized. At the end of assembly, the solid-support bound oligoribonucleotides were treated with 1:3 ethanol/ammonia (aq. 26%) solution (16 mL) at 55 °C for 5 h, in a sealed sterile vial diluted to twice the volume with sterile water and lyophilized. The crude sequences were dissolved in DMSO (4 mL) at 65 °C and treated with triethylamine trihydrofluoride (4 mL) at 65 °C for 2.5 h to remove the TBDMS groups. The resulting oligoribonucleotides were returned to RT, precipitated using *n*-butanol (24 mL), cooled to 0 °C for 20 min and centrifuged for 10 min at 5 °C. The resulting supernatant was decanted off and the formed pellet was re-suspended in *n*-butanol (24 mL) and the procedure repeated. Finally, the pellet was dissolved in water (20 mL) and lyophilized to yield the crude oligoribonucleotide. All oligoribonucleotides were purified by reverse-phase HPLC on a XBridge BEH column OBD (C18, 19 × 150 mm, 5 µm, 130 Å pore), using a gradient of acetonitrile in 100 mM triethylammonium acetate (pH 7.4). After HPLC purification, oligoribonucleotides were lyophilized and dissolved in phosphate buffer without the need for desalting. All oligoribonucleotides were characterised by electrospray mass spectrometry using a Waters Acquity LC-TOF-MS instrument in ESI-mode.

## Supplementary figures and tables

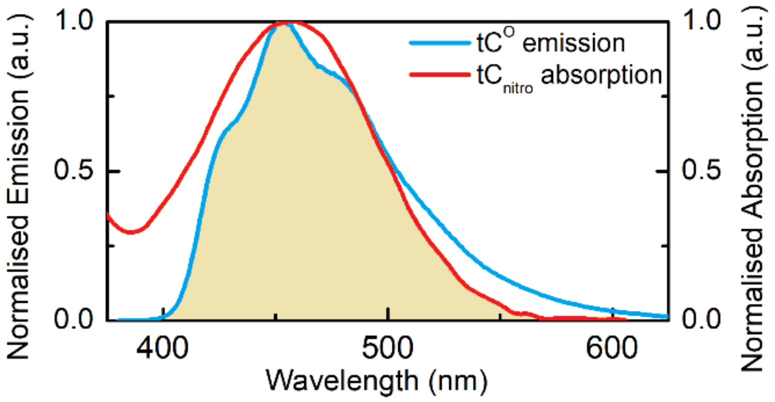


**Figure S1.** Spectral overlap of tC$^{O}$ emission and tC$_{nitro}$ absorption in double-stranded RNA[1]. The spectra are normalised at their long-wavelength maxima. Absorption maximum of tC$^{O}$ is at 370 nm and emission maximum at 456 nm. Absorption maximum of non-emissive tC$_{nitro}$ is at 454 nm. Fluorescence quantum yield of tC$^{O}$ is 0.22.

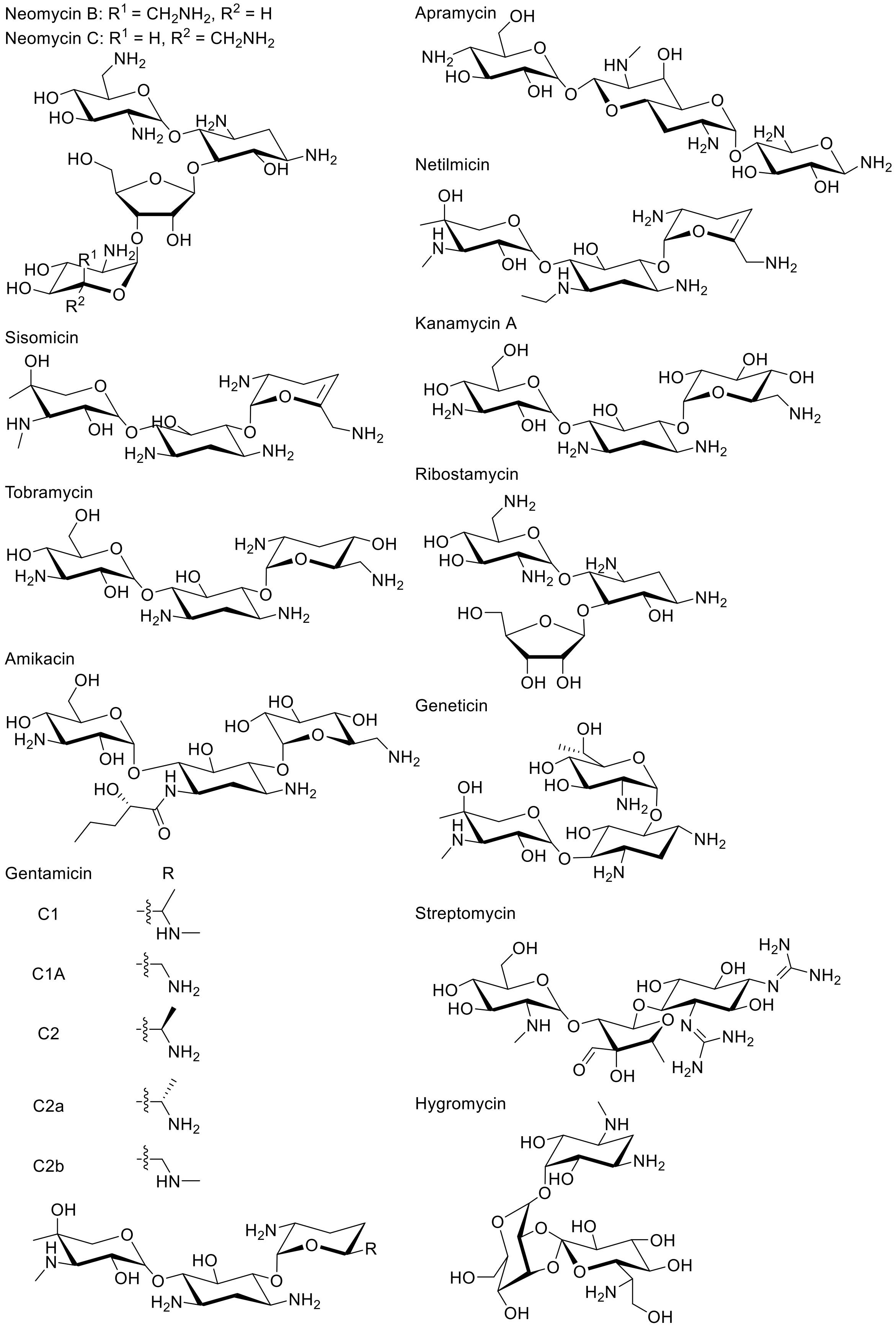


**Figure S2**. Structures of aminoglycosides used in this study.

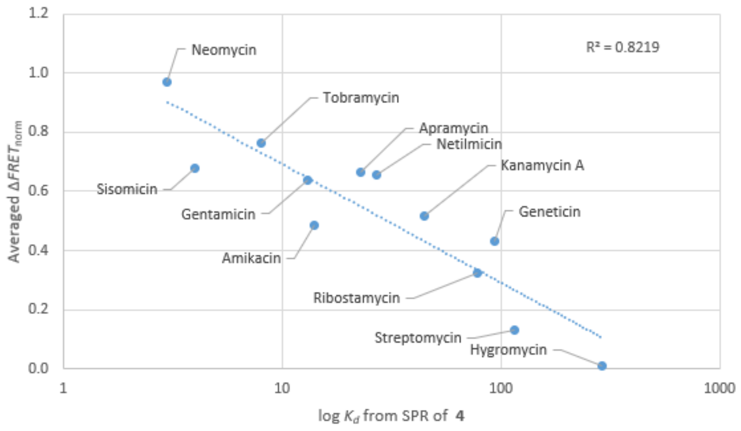


**Figure S3.** Correlation of log $K_d$ of **4** with the averaged $\Delta FRET_{norm}$.

**Table S1**. The sequences and mass of oligoribonucleotides synthesized in-house.

| ID | Oligoribonucleotide Sequence[a] | Expected avg. mass (Da) | Obtained avg. mass (Da) |
|---|---|---|---|
| **2** | 5′-CCG ACU GAU GUU GAC UGU UGA AUC UCA UGG CAA CAC CAG UCG G-3′ | 13752.31 | 13752.84 |
| **4** | 5´-biot-C6-CCG ACU GAU GUU GA**X** UGU UGA AU**Y** UCA UGG CAA CAC CAG UCG G-3′ | 14413.01 | 14412.05 |

[a] **X** denotes the fluorescent FRET donor $tC^O$ and **Y** denotes the non-emissive FRET acceptor $tC_{nitro}$.

**Table S2**. Acquired runs of SPR data for all aminoglycosides on oligoribonucleotides **3** and **4**. Every aminoglycoside was tested in triplicate. Immobilization levels for **3** were 430, 440 and 340 response units (RU), respectively, for the three different runs. Immobilization levels for **4** were 450, 540 and 370 RU, respectively.

| **Aminoglycoside** | ***K**d* **(M) of 3** | ***R**max* **of 3** | ***K**d* **(M) of 4** | ***R**max* **of 4** |
|---|---|---|---|---|
| Neomycin | 6.1E-06 | 65 | 3.4E-06 | 60 |
| | 7.6E-07 | 73 | 7.4E-07 | 82 |
| | 4.9E-06 | 102 | 3.8E-06 | 99 |
| Sisomicin | 5.3E-06 | 53 | 5.2E-06 | 58 |
| | 1.9E-06 | 64 | 2.1E-06 | 82 |
| | 4.4E-06 | 35 | 6.0E-06 | 43 |
| Tobramycin | 1.1E-05 | 57 | 9.9E-06 | 61 |
| | 3.3E-06 | 71 | 2.5E-06 | 88 |
| | 9.8E-06 | 44 | 1.1E-05 | 48 |
| Amikacin | 1.9E-05 | 85 | 1.6E-05 | 88 |
| | 1.2E-05 | 137 | 1.1E-05 | 154 |
| | 1.4E-05 | 44 | 1.6E-05 | 46 |
| Gentamicin | 1.7E-05 | 64 | 1.5E-05 | 69 |
| | 1.7E-05 | 98 | 1.0E-05 | 102 |
| | 1.5E-05 | 36 | 1.5E-05 | 43 |
| Apramycin | 1.9E-05 | 73 | 1.7E-05 | 80 |
| | 1.2E-05 | 118 | 1.4E-05 | 146 |
| | 3.6E-05 | 62 | 3.7E-05 | 69 |
| Netilmicin | 1.4E-05 | 67 | 1.2E-05 | 70 |
| | 3.0E-06 | 75 | 3.6E-06 | 94 |
| | 6.9E-05 | 59 | 6.6E-05 | 57 |

| | | | | |
|---|---|---|---|---|
| Kanamycin A | 5.1E-05 | 70 | 5.0E-05 | 72 |
| | 2.4E-05 | 92 | 2.0E-05 | 101 |
| | 5.8E-05 | 48 | 6.5E-05 | 54 |
| Ribostamycin | 7.4E-05 | 65 | 6.1E-05 | 63 |
| | 2.7E-05 | 77 | 2.1E-05 | 84 |
| | 1.7E-04 | 61 | 1.5E-04 | 57 |
| Geneticin | 9.9E-05 | 70 | 9.3E-05 | 72 |
| | 5.8E-05 | 84 | 3.7E-05 | 84 |
| | 1.5E-04 | 49 | 1.5E-04 | 53 |
| Streptomycin | 1.4E-04 | 89 | 1.2E-04 | 85 |
| | 5.7E-05 | 77 | 3.5E-05 | 75 |
| | 2.1E-04 | 73 | 1.9E-04 | 69 |
| Hygromycin | 2.8E-04 | 71 | 2.7E-04 | 75 |
| | 2.1E-04 | 104 | 1.9E-04 | 118 |
| | 3.9E-04 | 44 | 4.1E-04 | 48 |

**Table S3**. Binding stoichiometry of aminoglycosides to oligoribonucleotides **3** and **4**, calculated based on $R_{max}$ values and immobilization levels in Table S2 (average from SPR triplicate ± standard deviation).

| Aminoglycoside | MW | Stoichiometry of 3 | Stoichiometry of 4 |
|---|---|---|---|
| Neomycin | 614.7 | 4.7 ± 1.7 | 4.3 ± 1.7 |
| Sisomicin | 447.5 | 3.9 ± 0.7 | 4.3 ± 0.6 |
| Tobramycin | 467.5 | 4.3 ± 0.5 | 4.4 ± 0.5 |
| Amikacin | 585.6 | 5.0 ± 2.2 | 5.1 ± 2.0 |
| Gentamicin | 477.6 | 4.7 ± 1.8 | 4.6 ± 1.1 |
| Apramycin | 539.6 | 5.4 ± 1.4 | 5.7 ± 1.4 |
| Netilmicin | 475.6 | 4.9 ± 0.3 | 4.9 ± 0.3 |
| Kanamycin A | 484.5 | 5.0 ± 1.0 | 4.9 ± 0.6 |
| Ribostamycin | 454.5 | 5.3 ± 0.5 | 4.8 ± 0.2 |
| Geneticin | 692.7 | 3.4 ± 0.5 | 3.2 ± 0.2 |
| Streptomycin | 581.6 | 4.8 ± 0.5 | 4.3 ± 0.7 |
| Hygromycin | 527.5 | 4.7 ± 1.5 | 4.7 ± 1.2 |

**Table S4**. FRET efficiency change (Δ*FRET*) ± standard deviation (SD) when adding aminoglycoside to oligoribonucleotide **5** (FRET-labelled in the hairpin loop region). Oligoribonucleotide **6** was used as the donor-only reference.

| Aminoglycoside | $\Delta FRET_{15\ \mu M}$ | SD | $\Delta FRET_{90\%}$ | SD | $\Delta FRET_{norm}$ |
|---|---|---|---|---|---|
| Neomycin | 0.042 | 0.002 | 0.041 | 0.002 | 1.02 |
| Sisomicin | 0.023 | 0.004 | 0.040 | 0.006 | 0.59 |
| Tobramycin | 0.024 | 0.002 | 0.029 | 0.000 | 0.82 |
| Amikacin | 0.021 | 0.001 | 0.044 | 0.001 | 0.47 |
| Gentamicin | 0.011 | 0.001 | 0.025 | 0.005 | 0.44 |
| Apramycin | 0.017 | 0.002 | 0.026 | 0.001 | 0.64 |
| Netilmicin | 0.024 | 0.001 | 0.047 | 0.001 | 0.52 |
| Kanamycin A | 0.009 | 0.004 | 0.025 | 0.003 | 0.36 |
| Ribostamycin | 0.006 | 0.000 | 0.025 | 0.001 | 0.24 |
| Geneticin | 0.010 | 0.002 | 0.028 | 0.004 | 0.35 |
| Streptomycin | 0.004 | 0.002 | 0.034 | 0.001 | 0.11 |
| Hygromycin | 0.001 | 0.000 | -0.052 | 0.002 | 0.02 |

**Table S5**. FRET efficiency change ($\Delta FRET$) ± standard deviation (SD) when adding aminoglycoside to oligoribonucleotide **7** (FRET-labelled in the stem region). Oligoribonucleotide **8** was used as the donor-only reference.

| Aminoglycoside | $\Delta FRET_{15\ \mu M}$ | SD | $\Delta FRET_{90\%}$ | SD | $\Delta FRET_{norm}$ |
|---|---|---|---|---|---|
| Neomycin | 0.045 | 0.003 | 0.050 | 0.002 | 0.92 |
| Sisomicin | 0.041 | 0.000 | 0.054 | 0.000 | 0.77 |
| Tobramycin | 0.049 | 0.014 | 0.069 | 0.003 | 0.71 |
| Amikacin | 0.020 | 0.007 | 0.039 | 0.002 | 0.50 |
| Gentamicin | 0.040 | 0.005 | 0.048 | 0.003 | 0.83 |
| Apramycin | 0.037 | 0.003 | 0.053 | 0.001 | 0.67 |
| Netilmicin | 0.045 | 0.003 | 0.057 | 0.011 | 0.79 |
| Kanamycin A | 0.041 | 0.001 | 0.060 | 0.003 | 0.68 |
| Ribostamycin | 0.024 | 0.000 | 0.059 | 0.008 | 0.41 |
| Geneticin | 0.027 | 0.004 | 0.053 | 0.000 | 0.51 |
| Streptomycin | 0.008 | 0.003 | 0.055 | 0.005 | 0.15 |
| Hygromycin | 0.000 | 0.001 | -0.118 | 0.000 | 0.00 |

**Table S6**. Averaged normalized FRET efficiency change for the two FRET-labelled oligoribonucleotides, **5** and **7**, (*Averaged* $\Delta FRET_{norm}$), obtained by averaging their $\Delta FRET_{norm}$ values.

| Aminoglycoside | *Averaged* $\Delta FRET_{norm}$ |
|---|---|
| Neomycin | 0.97 |
| Sisomicin | 0.68 |
| Tobramycin | 0.77 |
| Amikacin | 0.49 |
| Gentamicin | 0.64 |
| Apramycin | 0.67 |
| Netilmicin | 0.66 |
| Kanamycin A | 0.52 |
| Ribostamycin | 0.33 |
| Geneticin | 0.43 |
| Streptomycin | 0.13 |
| Hygromycin | 0.01 |

**Table S7**. Aminoglycoside concentrations used to obtain 90% degree of complexation for the FRET experiment. Prepared from 10 mM stock solutions.

| Aminoglycoside | Concentration used to reach 90% degree of complexation in the FRET experiment (μM) |
|---|---|
| Neomycin | 29 |
| Sisomicin | 135 |
| Tobramycin | 134 |
| Amikacin | 250 |
| Gentamicin | 145 |
| Apramycin | 180 |
| Netilmicin | 350 |
| Kanamycin A | 330 |
| Ribostamycin | 750 |
| Geneticin | 550 |

| | |
|---|---|
| Streptomycin | 2400 |
| Hygromycin | 2300 |

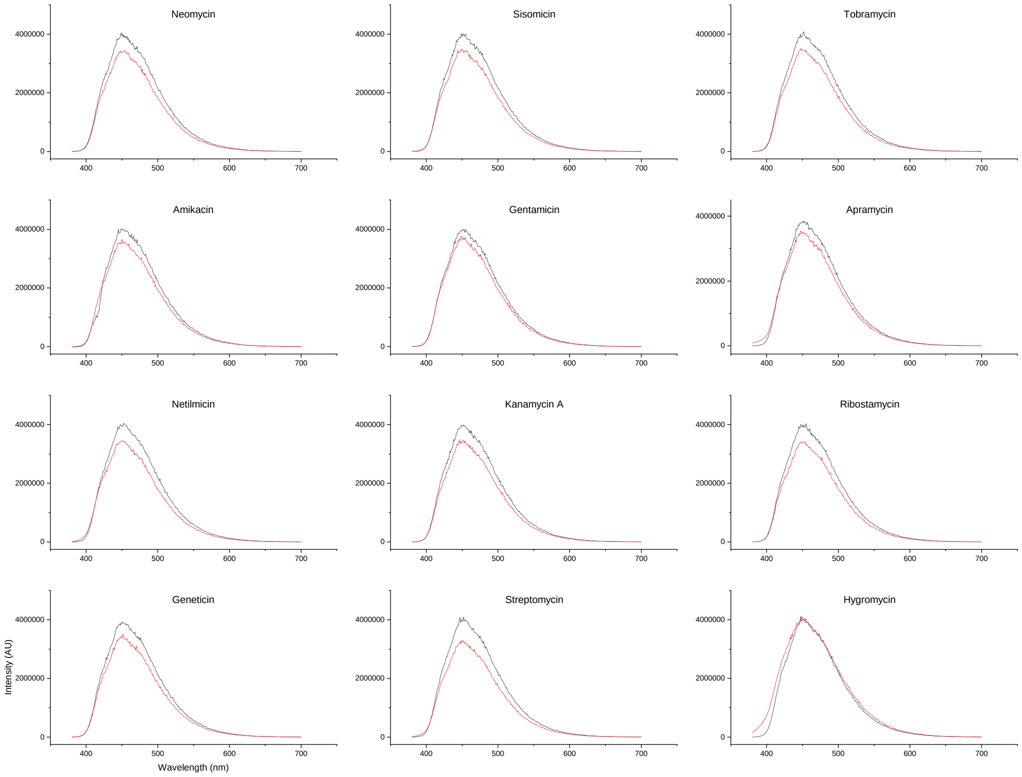


**Figure S4**. Emission spectra of pre-miR-21 **5** before (black) and after (red) addition of ligand for 90% degree of complexation.

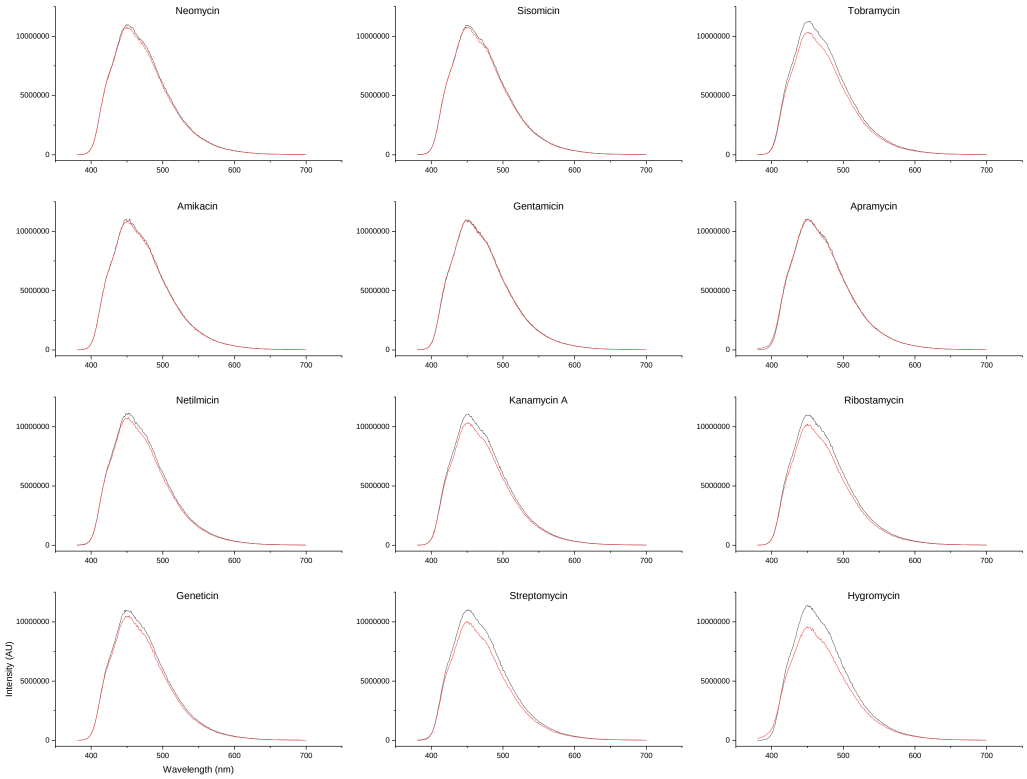


**Figure S5**. Emission spectra of pre-miR-21 **6** before (black) and after (red) addition of ligand for 90% degree of complexation.

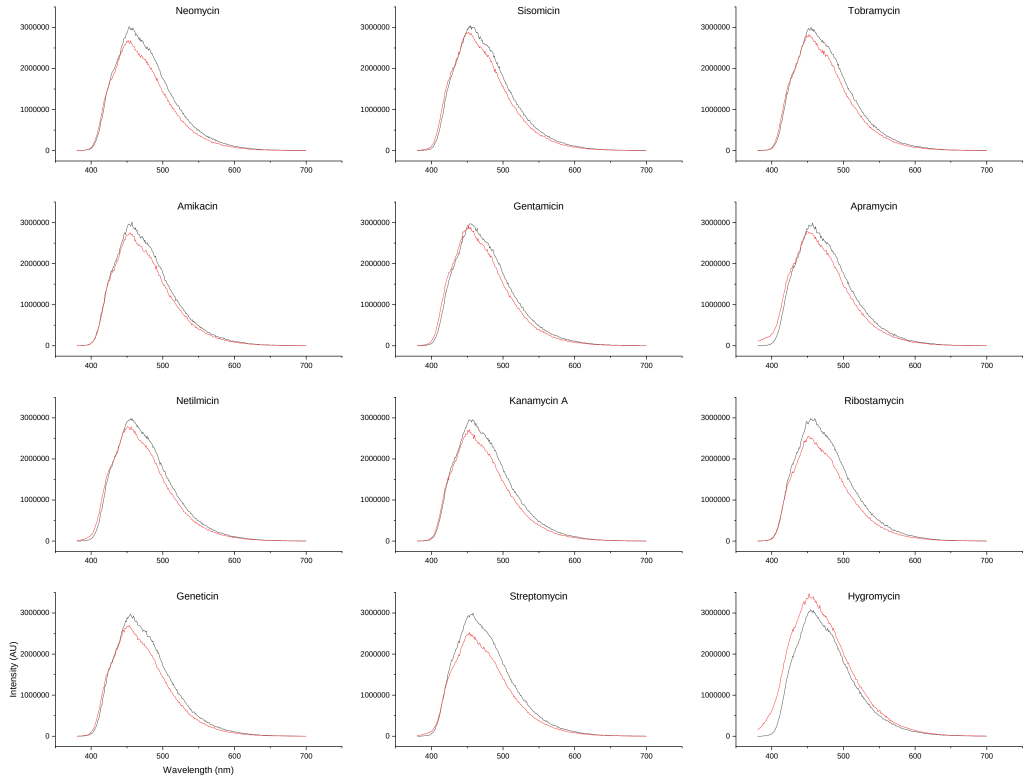


**Figure S6**. Emission spectra of pre-miR-21 **7** before (black) and after (red) addition of ligand for 90% degree of complexation.

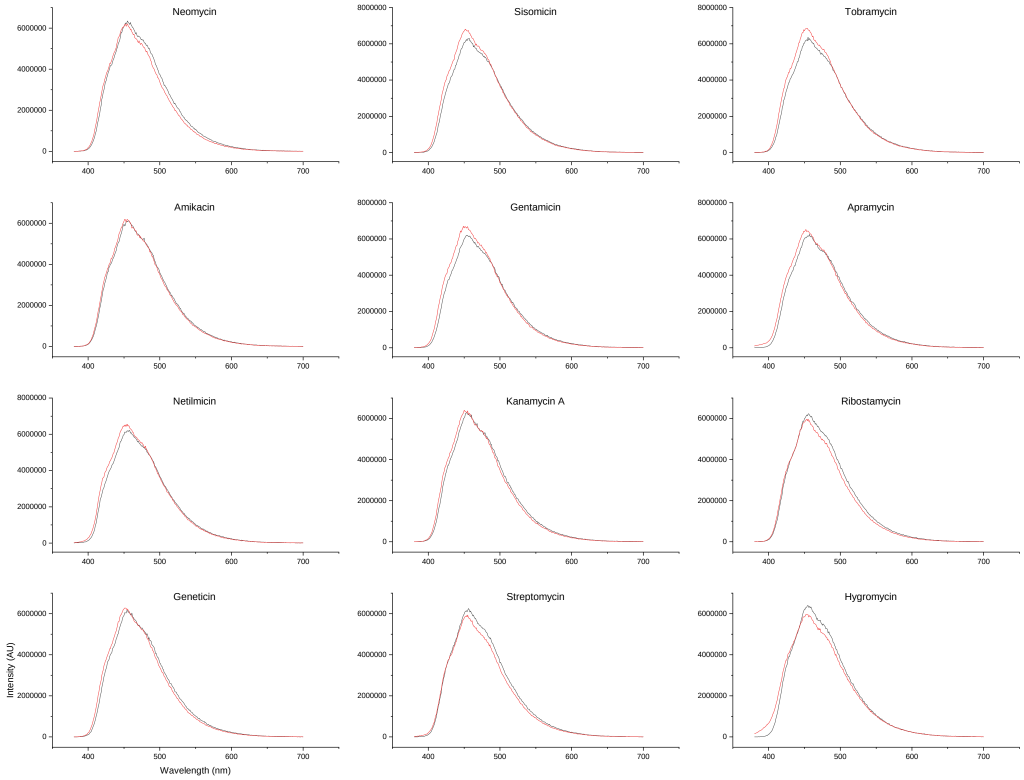


**Figure S7**. Emission spectra of pre-miR-21 **8** before (black) and after (red) addition of ligand for 90% degree of complexation.

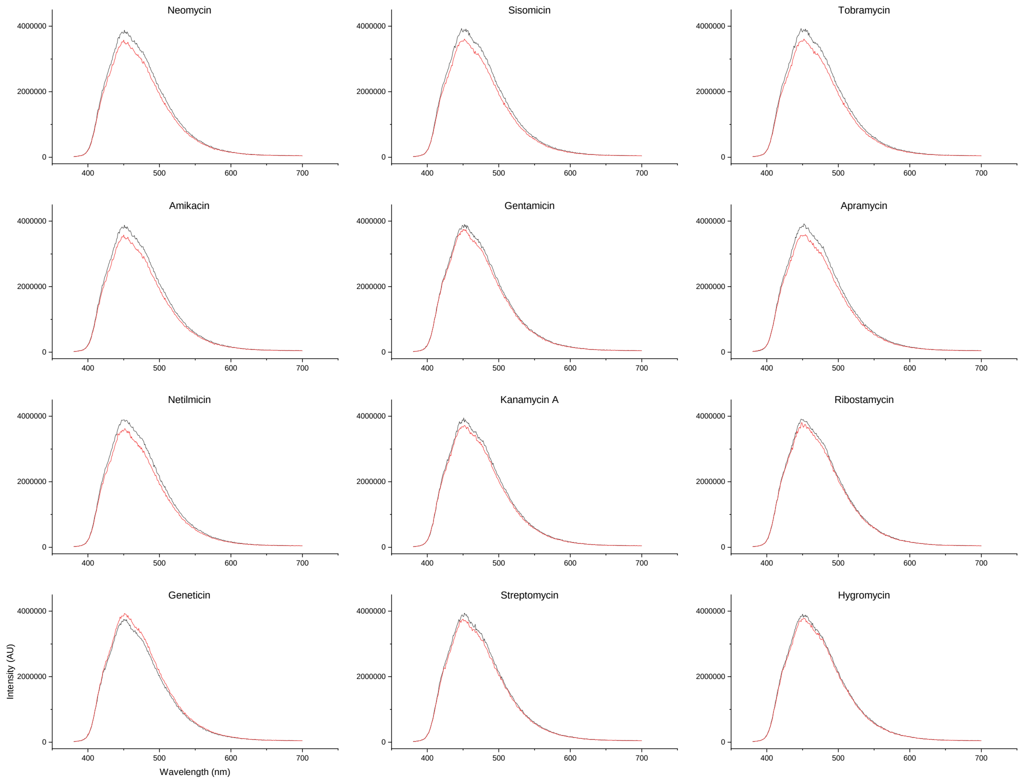


**Figure S8**. Emission spectra of pre-miR-21 **5** before (black) and after (red) addition of 15 μM ligand.

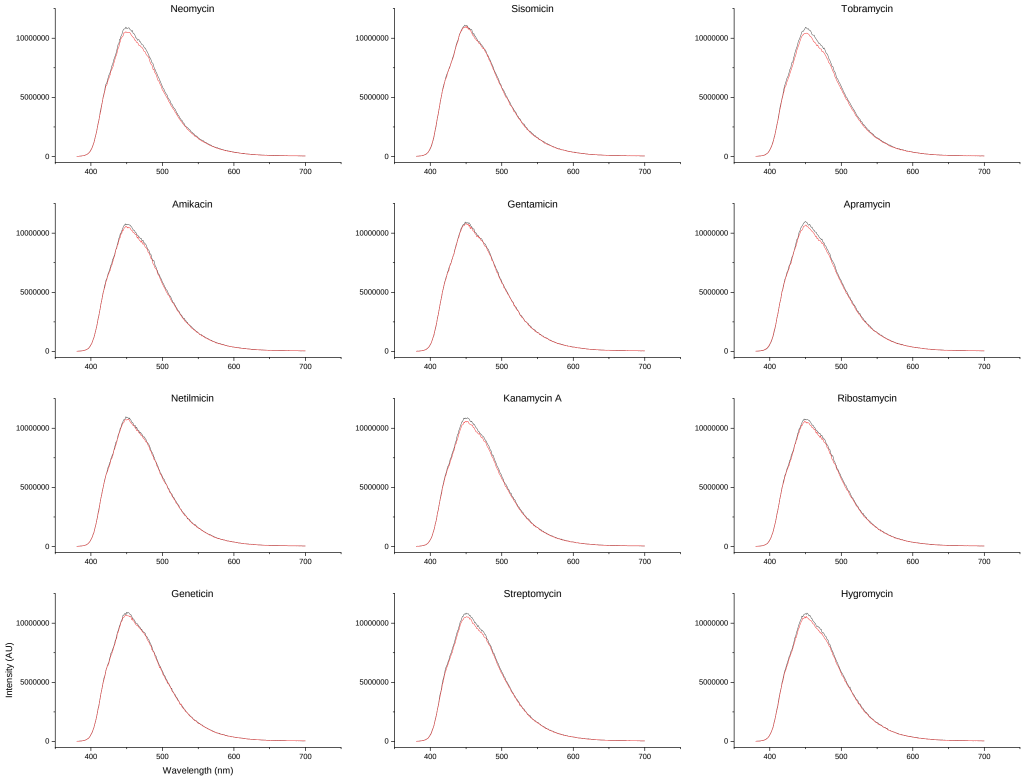


**Figure S9**. Emission spectra of pre-miR-21 **6** before (black) and after (red) addition of 15 μM ligand.

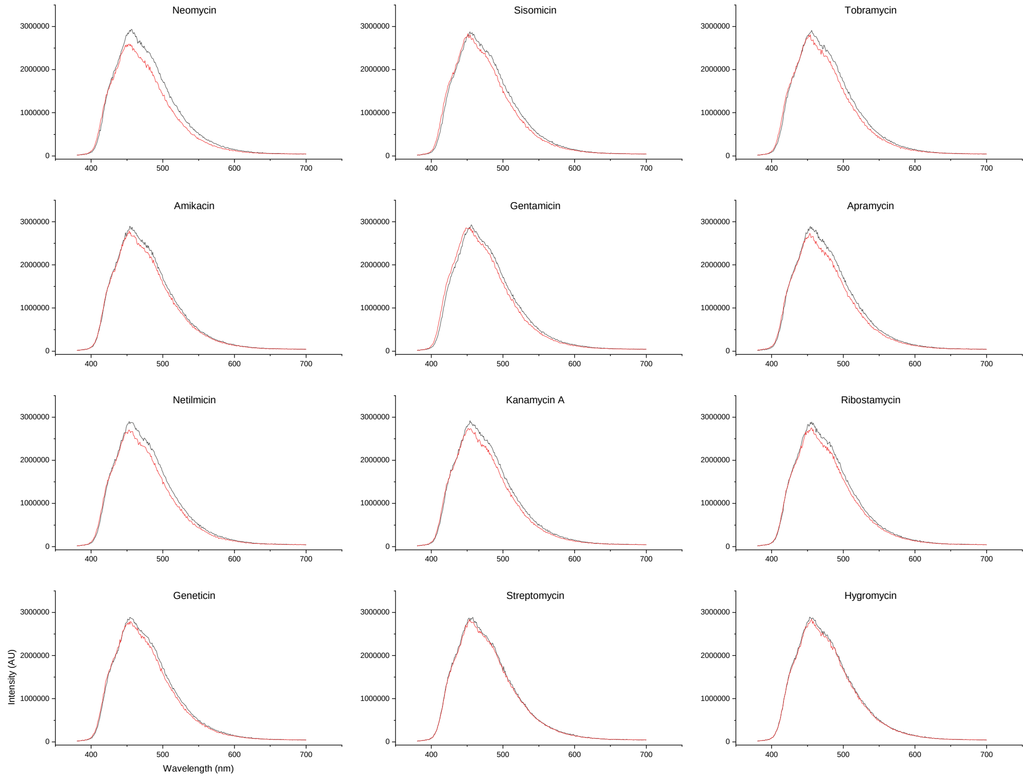


**Figure S10**. Emission spectra of pre-miR-21 **7** before (black) and after (red) addition of 15 μM ligand.

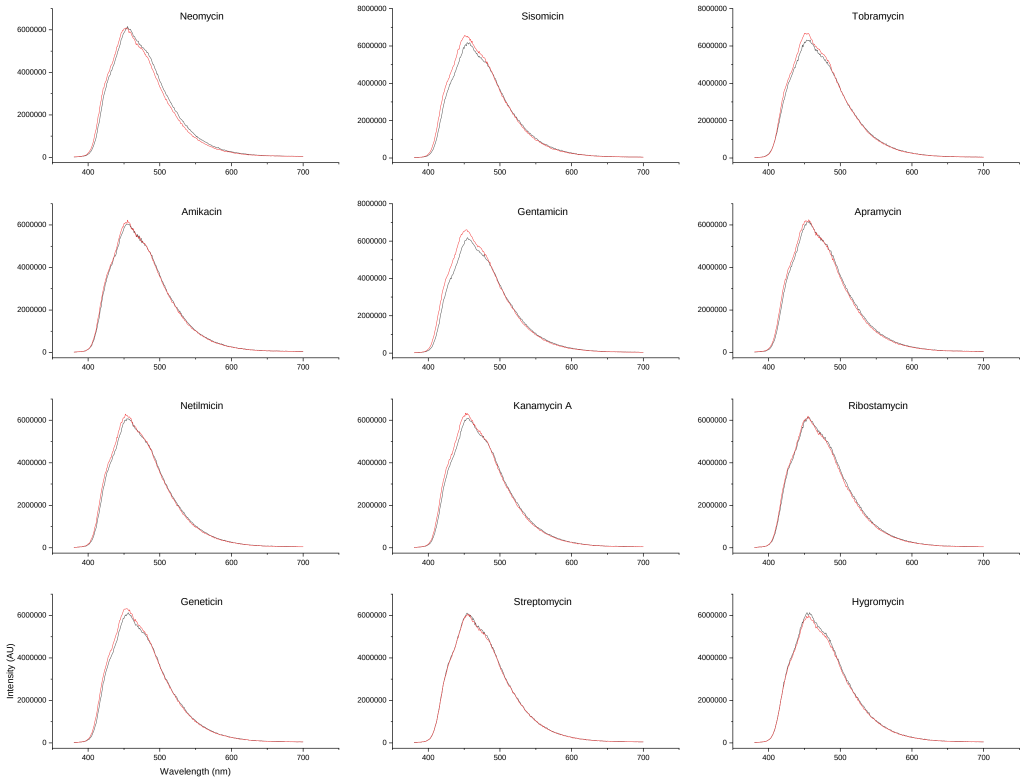


**Figure S11**. Emission spectra of pre-miR-21 **8** before (black) and after (red) addition of 15 μM ligand.

# References


1. Füchtbauer, A.F., Wranne, M.S., Bood, M., Weis, E., Pfeiffer, P., Nilsson, J.R., Dahlén, A., Grøtli, M. and Wilhelmsson, L.M. (2019) Interbase FRET in RNA: from A to Z. *Nucleic Acids Res.*, **47**, 9990-9997.
